# Aortic replacement in aorto-occlusive disease: an observational study

**DOI:** 10.1186/1471-2482-8-19

**Published:** 2008-10-31

**Authors:** Gareth Morris-Stiff, Samuel Ogunbiyi, Richard K Winter, Russell Brown, Michael H Lewis

**Affiliations:** 1 Department of Surgery, Royal Glamorgan Hospital, Llantrisant, Rhondda Cynon Taf, UK; 2 Department of Radiology, Royal Glamorgan Hospital, Llantrisant, Rhondda Cynon Taf, UK; 3 Health Solutions Wales, Cardiff, Cardiff, UK

## Abstract

**Background:**

For many patients with aorto-occlusive disease, where stent deployment is not possible, surgery remains the only treatment option available. The aim of this study was to assess the results of aortic reconstruction surgery performed in patients with critical ischaemia.

**Methods:**

All patients with critical ischaemia undergoing surgery during 1991–2004 were identified from a prospectively maintained database. Mortality data was verified against death certificate data. Demographic and clinical data were obtained from the clinical notes and the radiology database. Disease was classified as: type I – limited to aorta and common iliac arteries; type II – external iliac disease and type III combined aortic, iliac and infra-inguinal disease.

**Results:**

86 patients underwent aortic replacement surgery all of whom had critical ischaemia consisting of: type I (n = 16); type II (n = 37) and type III (n = 33). The 30-day mortality rate was 10.4%, the one-year patient survival was 80%, and the 1-year graft survival was 80%. At 2 years the actual patient survival was 73% and no additional graft losses were identified. All patients surviving 30 days reported excellent symptomatic relief. Early, complications occurred in 6 (7%) patients: thrombosis within diseased superficial femoral arteries (n = 4); haemorrhage and subsequent death (n = 2). Ten (14%) late complications (> 12 months) occurred in the 69 surviving patients and included: anastomotic stenosis (n = 3); graft thrombosis (n = 4), graft infection (n = 3). Four patients developed claudication as a result of more distal disease in the presence of a patent graft, and 1 patient who continued smoking required an amputation for progressive distal disease.

**Conclusion:**

Aortic reconstruction for patients with extensive aorto-occlussive disease provides long-standing symptomatic relief for the majority of patients. After the first year, there is continued patient attrition due to co-existent cardiovascular disease but no further graft losses.

## Background

The infra-renal abdominal aorta and iliac arteries are amongst the commonest sites for symptomatic stenotic atherosclerotic lesions [1]. Stenoses are most commonly identified in related to arterial bifurcation; of the aorta into common iliac arteries, and common into internal and external iliac vessels.

Aorto-occlussive diseases (AOD) may be classified into 3 subtypes: I – stenoses within the aorta and common iliacs; II – stenoses extending into the external iliac arteries; III – stenoses within the aorta, iliac vessels and common femoral arteries [2]. The typical distribution of AOD, as reported in the literature, is type III (65%) followed by type II (25%) and type I (10%). Patients with type I AOD are frequently asymptomatic due to the development of a network of collaterals, however, they can develop claudication within the proximal musculature of the buttock and thigh [3]. Type II AOD usually presents with buttock and thigh claudication whilst patients with type III disease typically present with intermittent claudication.

The development of balloon technology allowing percutaneous angioplasty and vascular stenting has meant that many patients with focal iliac lesions and short stenoses within the femoropopliteal artery catheter mediated treatment has become the gold-standard of care [4]. However, whilst some success has been reported for subgroups of patients with aortoiliac disease [5], for many patients with bilateral, multifocal or long-segment ileofemoral disease and for patients with additional femoral disease, these procedures are inappropriate and symptomatic patients are usually treated by means of surgical bypass of the disease segment. However, the results of surgical treatement of AOD as reported in the literature are far from perfect with major complications reported in 20–25% of cases including a 10–20% occlusion rate at 5 years and 25–30% occlusion by 10 years [6-14].

There is also a significant morbidity associated with aortofemoral grafting for occlusive disease as the majority of patients are arteriopaths with multifocal disease including cardiovascular and cerebrovascular circulations and are also frequently smokers leading to additional respiratory risk factors [15].

The aim of this study was to report the experiences of a single surgeon in the management of AOD not amenable to catheter-based therapies including short- and long-term complications, and patient survival rates.

## Methods

All patients undergoing surgery for AOD as a result of critical ischaemia during the period 1991 to 2004 were identified from a prospectively maintained vascular surgery database. Ethical approval for the study was granted by the institutional review board. Each patient complied with the definition of critical ischaemia namely: (1) more than two weeks of recurrent foot pain at rest that requires regular use of analgesics and is associated with an ankle systolic pressure of 50 mm Hg or less, or a toe systolic pressure of 30 mm Hg or less or (2) a non-healing wound or gangrene of the foot or toes, with similar haemodynamic measurements [16]. Only patients with confirmed aortoiliac occlusive disease not amenable to angioplasty or stenting were included and those with surgical disease limited to the ileofemoral vascular tree were excluded.

All patients with a chronic presentation who had been seen in the outpatient department had undergone a careful screening for the presence of cardiovascular risk factors including: hypertension; diabetes mellitus; hypercholesteolaemia; smoking; and a history of cardiovascular/cerebrovascular disease. In all cases where new risk factors were identified, appropriate modifications were implemented. Patients with an acute presentation could not benefit from such an approach. Individuals with ongoing or past symptoms of cardiovascular/cerebrovascular disease were assessed by a cardiologist and further investigations were implemented as indicated.

The vascular assessment included a full examination followed by ankle brachial pressure index (ABPI) and Doppler assessment. Angiography was performed to delineate the extent and distribution of disease. During the course of the study, this evolved from digital subtraction to magnetic resonance angiography.

Prior to operation all patients were reviewed in a multidisciplinary meeting with at least 1 vascular interventional radiologist at which none of the patients were deemed suitable for balloon angioplasty or intravascular stenting. The extent of AOD was classified by a vascular radiologist in each case. All patients had a minimum 2-year potential follow-up.

Demographic details, cardiovascular risk factors and follow-up data including postoperative complications were identified from patient case notes.

All operations were performed by a single surgeon and followed the same operative technique. The standard operative procedure consisted of a midline laparotomy through which the small bowel was retracted allowing access to the aorta. Having confirmed the preoperative distribution of disease within the intra-abdominal vascular tree, the lumen of the aorta was partially occluded using a Satinsky arterial clamp. An arteriotomy was performed and the proximal end of an appropriately-sized polyester graft was sutured in place end-to-side. The flow was then restored whilst dissection of the femoral arteries continued. The limbs of the graft were then tunnelled beneath the inguinal ligaments as an anatomical bypass and anastomosed onto suitable segments of femoral artery, usually the common femoral. In 28 of 38 bypasses performed for Type III disease a femoral disobliteration or profundoplasty was performed and in 6 cases a femoropoliteal bypass was constructed during the same anaesthetic.

Post-operatively, all patients were followed in the vascular outpatient clinic where a clinical assessment was performed with subsequent imaging studies as indicated. Secondary prevention measures based upon minimising the cardiovascular risk profile were continued postoperatively.

Mortality data was obtained form the hospital patient information system and verified against CEPOD (confidential enquiry into perioperative deaths) returns and to death certificate returns to Health Solutions Wales.

A Kaplan-Meier analysis was performed to examine the survival of patients undergoing surgical bypass using MedCalc 9.4.2 (^® ^MedCalc, Frank Schoojans, Mariakerke, Belgium).

## Results

During the period of the study, 86 patients (47 males and 39 females) with critical ischaemia underwent aortobifemoral bypass for AOD with a mean patient age was 60 years (SEM: 1 year; range: 40–80). All patients had rest pain not responding to analgesia and all were classified as ASA (American Society of Anaesthesiologists) grade II (n = 39) or III (n = 47). All patients had significant cardiovascular disease and many had a history of respiratory disease or were smokers as summarised in Table 1.

**Table 1:** Cardiovascular risk factor profiles of patients undergoing aortic reconstruction for AOD.

**Cardiovascular Risk Factors**	**Type I** **(n = 18)**	**Type II** **(n = 40)**	**Type III** **(n = 28)**
Hypertension	8 (44%)	35 (88%)	23 (82%)
Hypercholesterolaemia	10 (56%)	28 (70%)	19 (67%)
Diabetes Mellitus	1 (6%)	5 (13%)	4 (14%)
Angina	4 (22%)	18 (45%)	13 (46%)
Myocardial Infarct	1 (6%)	7 (18%)	8 (29%)
Transient Ischaemic Attack	0 (0%)	4 (10%)	4 (14%)
Cerebrovascular Accident	0 (0%)	2 (5%)	2 (7%)
Smoking (Current or Ex-smoker)	13 (72%)	34 (85%)	22 (79%)
Respiratory Disease	5 (28%)	15 (38%)	11 (39%)

The most common distribution of AOD was type II (n = 37; 43%) followed by type III (n = 33; 38%) then type I (n = 16; 19%).

The complications are summarised in Table 2. Early complications, within 12 months of surgery, were noted in 6 cases (7%) and consisted of 4 cases of thrombotic occlusion of a diseased but previously patent superficial femoral artery distal to the graft and 2 cases of fatal peri-operative haemorrhage. All 4 episodes of thrombosis occurred in patients with Type III disease in which the extent of femoropopliteal disease had not been appreciated preoperatively due to the poor inflow. All 4 patients underwent thrombectomy and distal reconstruction and no limbs were amputated.

**Table 2:** Complications experienced following aortic reconstruction for AOD.

**Complication**	**Number of Patients (%)**
***Early***	6/86 (7%)
Thrombosis within diseased SFA	4
Fatal haemorrhage	2
***Late ***	10/69 (14%)
Graft stenosis	3
Graft thrombosis	4
Graft infection	3

The 2 patient deaths occurred in patients of ASA grade 3 who had been admitted as emergencies with critical ischaemia and borderline viability as a result of acute deterioration of their chronically ischaemic limbs. Both patients suffered significant bleeding following reperfusion due to the development of a coagulopathy. This in turn lead to peri-operative myocardial ischaemia then subsequent cardiac arrest during the early post-operative phase.

Late complications, occurring after 12 months, were seen in 10 of 69 (14%) surviving patients. The complications consisted of: 3 episodes of graft stenosis, 4 cases of graft thrombosis; and 3 cases of late graft infection. All 3 episodes of graft stenosis occurred at the distal anastomosis and were due to intimal hyperplasia at the anastomotic site. In each case, balloon angioplasty was successful in dilating the stenosed segment and surgical exploration was not required.

In addition 4 patients with no prior history of calf claudication developed new symptoms. In these 4 cases, investigation revealed progression of disease in distal vessels despite good quality inflow and patent grafts. Only 1 patient has required an amputation, this being a smoker with Type III AOD who continued his habit after a good initial result from surgery and underwent a below knee amputation 3 years later due to progressive distal disease.

**Figure 1:**
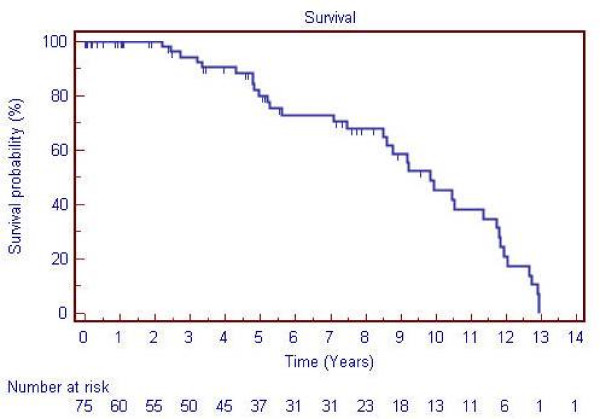
Kaplan-Meier survival curve for patients undergoing aortic reconstruction for AOD.

The 30-day mortality was 10.4%, with an actual 1-year patient and graft survival of 80%. This early mortality included: the 2 cases related to haemorrhage already detailed; 2 of multi-organ failure and 5 related to myocardial infarction. At 2 years follow-up, there were an additional 6 deaths due to cardiovascular disease but no further graft losses were identified. If the deaths within 1-year are excluded the median survival of those patients who subsequently died during follow-up is 4.65 years. The survival of patients undergoing bypass is summarised in Figure 1.

During the overall period of follow-up ranging from 1 to 13 years, 53 of 86 patients died. The causes of death are summarised in Table 3. The vast majority, 41/53 were cardiovascular in origin either cardiovascular (n = 35) or cerebrovascular (n = 5).

**Table 3:** Causes of death following aortic reconstruction for AOD.

**Cause of Death**	**Number of Patients**
Cardiovascular	35
Respiratory	5
Cerebrovascular accident	4
Carcinomatosis	3
Sepsis	2
Haemorrhage	2
Diabetes mellitus	1
Perforated peptic ulcer	1

## Discussion

The primary finding of the study was that aortobifemoral grafting for AOD can be performed with acceptable perioperative morbidity and morality, in a group with significant comorbidity, and with it providing excellent symptomatic relief for the vast majority of patients. On the surface, some aspects of this series may appear inferior to many of the large published series [6-14], however, a number of factors need to be considered. All the previous series, the most recent of which was 1992 describe an era before catheter based therapies were available or at best when the technology was not in widespread use. They therefore include a number of younger and fitter patients with less extensive cardiovascular and less advanced peripheral vascular disease which in many cases may have been amenable to dilatation or stenting. We have had a full interventional radiology service at our disposal since the first patient was treated and all vascular patients have been discussed in a surgical-radiology conference with the radiological interventionalist prior to surgery. Therefore numbers were significantly smaller than published series, patients had more advanced disease at presentation and consequently had a high risk cardiocvascular profile.

Furthermore, several of the published series provide no or incomplete data about the disease distribution and severity, and in many case there is a wide variation in the indication for operation from moderate claudication to critical ischaemia. The variation in severity of cardiovascular disease between this study and other published studies is typified by the data from the Leiden group which notes comparable rates of long-term mortality from cardiovascular and malignant causes [14]. In the current series, cardiovascular causes including cardio- and cerebro-vascular causes accounted for 74% of deaths indicating that our patients had more advanced cardiovascular disease.

Interestingly, van den Akker [14] also provided a breakdown of their results according to patient symptoms and note that results, including patient survival and graft thrombosis rates, were significantly poorer for patients with critical ischaemia. This observation was also been noted by Crawford *et al*. [12] who reported significantly worse early (6% *versus *3%) and late mortality (55% *versus *39%) rates in patients with rest pain when compared with claudication alone. When this is taken into consideration, together with the fact that many the Houston patients may have been suitable for angioplasty then the 10.4% 30-day mortality in our series appears far more acceptable.

A further interesting observation was the fact that the distribution of disease in the current study was significantly different to that reported by other centers. Whilst Brewster and Darling [9] reported relative percentages for Types I-III of 13:21:66, with a significantly higher proportion of Type III, Szilagyi and colleagues [13] noted the opposite with a ratio of 65:21:14. Patients with Type II and III disease have a higher prevalence of: diabetes; hypertension; cardio- and cerebrovascular disease, and so it is not surprising that these patients have a poorer outcome in relation to mortality and disease recurrence/progression[15].

Interestingly for the cohort surviving a year, there were a further 6 deaths between 1- and 2-years, however, there were no additional graft losses in this period. All the deaths in this group were due to cardiovascular causes suggesting that aggressive management of cardiovascular risk factors mat be of significant benefit for this cohort of patients.

Another surgical alternative for high risk patients with critical ischaemia would be axillobifemoral grafting [17]. However, when this procedure is performed in high risk patients with critical ischaemia the results are significantly worse than for aortobifemoral grafting with mortalities of up to 18% and graft patencies of 33–85% reported. These are significantly worse than for axillobifemoral and so the procedure has limited value for patients fit enough to undergo aortobifemoral grafting.

A recent article by Upchurch and colleagues examined the effects of introduction of 'new technology' on management of aorto-iliac occlusive disease. During the period 1996 – 2000 they noted an 850% increase in stenting and a 15.5% reduction in bypass [18] and an overall significant increase in the number of procedures performed.

Unfortunately, no data was supplied as to the radiological distribution and severity of the disease or patient symptoms prior to intervention. Furthermore it did not look at outcomes in terms of complications, recurrent symptoms or 30-day or longer mortality. Interestingly, the majority of procedures were performed by radiologists. Whilst in hospital mortality rates were higher following bypass and duration of stay longer, it is difficult to directly compare such results and come up with valid conclusions as to the role of stenting as there was no means of comparing the 2 populations. To-date no randomized controlled trial has compared the two modalities. One of the major concerns addressed by those commenting on the paper were the training issues with reduced numbers of operations performed and management of vascular disease being drawn away from surgeons.

There has been considerable debate since the conception of surgical management for AOD which was recently summarized by Rutherford [19] and include: endarterectomy versus bypass; aorto-bisiliac versus aorto-bifemoral; end-to-side versus end-to-end; role of adjunctive profundoplasty; staged versus concomitant distal bypass; and axillo-femoral versus aaorto-bifemoral. In the authors experience almost all cases require bypass rather than simply endarterectomy with end-to-side proximal and bifemoral distal anastomoses with profundoplasty performed on an as required basis. This reduces the duration of the operation and hence the ischaemic time so limiting the potential ischaemia-reperfusion injury. Femoropopliteal bypass is only performed in selected basis at the primary operation or if there is no run off and axillofemoral grafts are limited to extremely high risk patients or those with hostile abdomens.

More recently, other options have been reported which may take over the mantle from the traditional open aortofemoral bypass including: hand-assisted laparoscopic [20]; totally laparoscopic [21]; robot-assisted bypass [22] and endografting [23]. Each method has its proponents who report potential benefits over open surgery but none have so far been compared in the setting of a randomized controlled trial. At present, although indications are fewer, aortofemoral bypass remains the gold-standard operation for patients with significant symptoms from AOD that is not amenable to catheter-based treatment.

## Conclusion

Aortic reconstruction provides long-standing symptomatic relief for the majority of patients with extensive aorto-occlussive disease for whom stenting is not feasible. However, the continued atrition in patient survival beyond the first postoperative year due to co-existent cardiovascular disease suggests that comprehensive cardiovascular risk factor management is required in these patients.

## Competing interests

The authors declare that they have no competing interests.

## Authors' contributions

MHL was responsible for the concept and performed the operations, SO assisted with data collection, RKW reviewed the radiology, RB provided long-term follow-up data and assisted with analyses. The paper was written by GMS with specialist contributions from RKW, RB and MHL. All authors approved the final version.

## Pre-publication history

The pre-publication history for this paper can be accessed here:


